# New Zealand radiation therapists’ perceptions of peer group supervision as a tool to reduce burnout symptoms in the clinical setting

**DOI:** 10.1002/jmrs.398

**Published:** 2020-05-20

**Authors:** Gay Dungey, Hazel Neser, Dalice Sim

**Affiliations:** ^1^ Department of Radiation Therapy University of Otago Wellington New Zealand; ^2^ University of Otago Wellington New Zealand

**Keywords:** Burnout, oncology, peer‐supervision, radiation therapists, support

## Abstract

**Introduction:**

Research indicates that radiation therapists (RTs) are at risk of burnout and that there is a lack of evidence on effective coping strategies for managing work‐related stressors within this workforce. Peer group supervision (PGS) is a useful tool in assisting staff to manage stress in the clinical setting, improve reflective practice and provide support. The aim of this research was to investigate New Zealand (NZ) RTs’ perceptions of participating in PGS.

**Methods:**

In‐service training on PGS was offered to all RT centres in NZ, and five of the nine centres agreed to partake in PGS. Participants anonymously completed the same online questionnaire, six months apart. The questionnaire consisted of the Clinical Supervision Evaluation Questionnaire (CSEQ), an open‐ended question and demographics. The CSEQ asks participants to indicate their agreement with 14 statements related to Purpose, Process and Impact of PGS.

**Results:**

Overall, 71 and 48 participants completed the first and second surveys, respectively. In contrast to previous studies, this study found that confidence in practice, team support and group safety were valued by participants. This was supported by the qualitative data that revealed four themes: supportive groups, time out to reflect, organisational barriers and group process issues. RTs with one to five years’ experience were more likely to structure their meetings, understand the purpose of the meetings and had clearer expectations of the group process.

**Conclusions:**

PGS may address burnout for RTs with one to five years’ experience. This group of RTs feel patient‐related matters can be discussed openly during PGS, and PGS appears to be helping to improve their practice and reduce stress. More experienced RTs appear to be using the groups as a ‘professional support group’, rather than ‘peer supervision’, as a strategy for managing organisational stressors associated with burnout.

## Graphical abstract

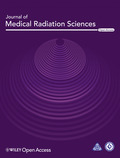

## Introduction

Over the last decade, several international studies indicate that radiation oncology workers including radiation therapists (RTs) are exposed to unique occupational stressors that put them at risk of burnout, such as treating dying patients, perceived poor management and a lack of career progression.^1^, ^2^, ^3^, ^4^, ^5^ According to the Maslach Burnout Inventory (MBI),^6^ burnout involves physical, mental and emotional exhaustion due to long‐term involvement in emotionally demanding situations. The first stage is emotional exhaustion where individuals feel emotionally overwhelmed by the demands of others. The second stage, depersonalisation, occurs by inappropriately attempting to cope with exhaustion and is characterised by feelings of detachment and dehumanisation. The final stage is a decreased sense of personal accomplishment and is associated with feelings of inadequacy, personal failure and poor professional self‐esteem.^6^

A national New Zealand (NZ) study found that radiation oncology workers experience high levels of occupational stress (such as excessive workloads, lack of recognition and professional development opportunities) and significantly high levels of burnout.^3^ Their burnout scores exceeded the MBI normative mean scores for medical workers, and the NZ scores exceeded those from previously published studies investigating burnout within the radiation oncology context.^3^ Findings from this NZ study revealed contradictory results. While patient stressors contributed positively to personal accomplishment and job satisfaction, they also caused high levels of emotional exhaustion and depersonalisation. Overall, addressing patient‐centred challenges was very rewarding. Participants discussed that patient‐centred challenges did not affect them as much as organisational challenges and that, particularly in the case of more experienced staff, patient challenges gave them a sense of personal accomplishment, contributing to job satisfaction.^3^ Participants indicated that it was organisational issues such as lack of career progression and poor management that detrimentally affected their well‐being rather than patient‐centred ones.^3^ These results were consistent with a similar Australian study that showed that Australasian oncology health professionals enjoyed providing care to patients but that this could sometimes compromise their well‐being and be a contributing factor to burnout.^8^

Findings from several studies highlight the need to support RTs and other oncology health professionals in order to prevent burnout and staff turnover.^2^, ^3^, ^4^, ^8^, ^9^ These issues need addressing because of increasing demands for cancer care within NZ and internationally. Given the unique occupational stressors of working in oncology, it is important that staff have opportunities to access support and tools to effectively manage their stress.^3^, ^4^, ^10^ These issues are also found in the NZ RT workforce. A NZ study that investigated how RTs manage their stress found that staff utilised a limited range of coping strategies – mainly socialising and exercise.^7^ Very few RTs made use of a nationwide confidential employment assistance programme (EAP), and even fewer had accessed professional clinical supervision as RT centres do not routinely provide funding for supervision sessions.^7^

RT staff development has traditionally focussed on enhancing technical skills rather than the interpersonal aspects of their patient and self‐care which has been attributed to: high workloads, clinical demands and organisational pressures.^11^ One way that time‐poor healthcare organisations can upskill RT staff on interpersonal aspects of their work can be through peer group supervision (PGS), sometimes described in the nursing literature as reflective practice groups.^12^ This is also a cost‐effective way of helping staff with work‐related stressors.^11^, ^12^, ^13^, ^14^ PGS allows peers of equal status, in groups of four to six, to focus on developing interpersonal skills to manage challenging clinical situations, workplace stress, emotional and ethical dilemmas.^13^ This differs from more traditional forms of counselling/clinical supervision in that it does not require the presence of a qualified expert to facilitate the process. Group members bring an issue for supervision to the group, and an agenda is set. Each group member then takes a turn as the supervisee, and the others collectively become the supervisor. The group uses a range of highly structured group processes for supervision, and the session ends with a final review in order to increase the group's effectiveness and ensure that members leave ‘intact’.^13^, ^14^ There is always a peer facilitator/leader assigned who safeguards the process to ensure the group sticks to the contract and maintains the focus of the supervisory process.^13^ If the structured process is not adhered to, groups could lack structure and degenerate into gossip, gripe and chat sessions or discussion groups. This can lead to mistrust and safety issues within the groups and/or individuals dominating and others becoming passive.^13^, ^15^

Evaluations of PGS in the allied health and nursing settings have established that staff report improved practice and patient care.^15^, ^16^, ^17^, ^18^ To the authors’ knowledge, there are no publications involving RTs participating in PGS. The aim of this research, therefore, was to trial and investigate NZ RTs’ perceptions of participating in PGS.

## Methods

A purposive sampling approach was used in order to target RTs. According to the authors’ knowledge, this is an under‐researched group in the field of PGS and little is known on how effective PGS would be in the RT arena. At the end of 2016, all nine radiation therapy centres in NZ were invited via email to have a face‐to‐face introductory in‐service to PGS. Seven centres accepted the in‐service invitation. The in‐services took place from December 2016 to February 2017. They involved one of the researchers travelling to each of the seven centres to present the concept and structure of PGS to the RT staff. At the same time, this study was also outlined. After the in‐service sessions, five centres agreed to trial PGS in their clinical setting. The decision to participate was voluntary. RT staff in each of the centres volunteered to participate in the PGS meetings. In mid‐2017, the New Zealand Coaching and Mentoring Centre facilitated two formal PGS workshops at two different cities in NZ. Representatives from the five centres attended one or other of the training workshops.

### Study participants

Following ethical approval from the University of Otago Ethics Committee (reference D17/374), all RTs participating in PGS, across the five centres, were invited to participate anonymously in two online questionnaires using SurveyMonkey™. The questionnaires were distributed, with participant information sheets, in June 2018 and January 2019 (nine months and 16 months after the formal PGS training, respectively). Repeating the questionnaire enabled us to detect meaningful factors (should they occur), while holding other variables (e.g. age and gender) constant. The decision to participate in the questionnaires was deemed as consent.

In June 2018, there were 109 RTs across the country participating in PGS; by January 2019, participation had dropped to 99 RTs. Reduced participation was influenced in part by parental leave, taking up new positions in other centres not participating in PGS, and moving overseas.

### The evaluation tool

The evaluation tool included quantitative and qualitative elements. Quantitative data were obtained from the first section of the questionnaire, which utilised the Clinical Supervision Evaluation Questionnaire (CSEQ). This is a 14‐item validated evaluation tool developed by Horton and colleagues to evaluate group supervision for speech therapists.^18^ Since then it has been found to have reliability and validity for group supervision in other allied health professions.^16^, ^17^ The CSEQ asks participants to indicate their agreement (on a 5‐point Likert scale) with 14 statements related to Purpose (three questions), Process (five questions) and Impact (six questions) of supervision. The CSEQ format used in this study was slightly modified with the permission of its authors; for example, the name of the activity being evaluated was changed to ‘Peer Group Supervision’.

Qualitative data were collected from the second section of the questionnaire, which included one optional, open‐ended question where participants could write free‐flowing text. This question asked participants what else they would like to say about their experience with PGS and enabled us to provide further insight into the quantitative data.

The third section collected the participants’ demographic data. Specifically, this was gender, ethnicity, age and years practising as an RT (excluding periods of absence of greater than six months).

### Data analysis

SPSS version 24 for Windows (IBM Corporation, Armonk, NY, USA) was used to describe the participants and their responses to the questionnaire. To compare our sample to previous samples in Horton et al.’s study,^18^ an exploratory factor analysis (maximum likelihood extraction and Oblimin rotation of Kaiser Normalisation) was undertaken requesting three factors: purpose, process and impact.

An inductive thematic analysis approach to the open‐ended responses from each survey was undertaken to provide complementary information to the quantitative data. These data were thick (quantity) but not rich (quality). Open‐ended responses were read and re‐read independently by two researchers (RT and an academic researcher) in order to develop codes. Initial coding was further refined through re‐reading responses independently and checking for consistency across the data set. The researchers then compared and discussed their independent coding and further refined codes. From this discussion, categories and themes emerged which were then discussed with the third researcher until consensus was reached. This researcher was not involved in data collection and was not an RT. Methodological triangulation was reached in this way to gain reliability of data analysis. No new themes emerged from the open‐ended responses in the second survey. To ensure reliability, the same survey was administered twice.

## Results

Overall, 119 participants completed the two surveys: 71 and 48 participants completed the first and second surveys, respectively. The sample in both surveys consisted of mostly New Zealand European females, aged between 20 and 39 years, who had between one and 15 years of RT clinical experience (Table 1). This is reflective of the general demographics of the New Zealand radiation therapy workforce.^3^, ^18^

**Table 1:** Participant characteristics.

	Survey 1 *n = 71*	Survey 2 *n = 48*
Gender		
Male	5	4
Female	66	44
Ethnicity		
NZ European	54	37
Maori	1	3
Samoan	1	0
Cook Island Maori	1	1
Chinese	3	2
Indian	1	1
Other	12	8
Age		
20–29 years	22	15
30–39 years	27	17
40–49 years	16	9
50–59 years	5	6
60+ years	1	1
Years of practice		
0–5 years	17	11
6–10 years	12	9
11–15 years	22	9
16–20 years	11	5
21–25 years	5	7
26–30 years	1	2
31+ years	3	5

The surveys were completed anonymously, so that it is unknown who participated in each survey. Therefore, it is not possible to compare the means of each survey to identify whether there were any changes in the perceptions of PGS after an in‐service presentation on initial findings from Survey 1 to participating RT centres. It was also not possible to analyse the data by ethnicity and gender as there were insufficient numbers of participants who were from ethnic minority backgrounds and/or who were male to make any meaningful comparisons.

A factor analysis with maximum likelihood extraction and Oblimin rotation of Kaiser Normalisation was undertaken. Three factors were requested. The results of this factor analysis produced different factors from Horton et al.’s ^18^ study, which identified purpose (Questions 1,9,14), process (Questions 2,3,5,10,13) and impact (Questions 4,6,7,8,11,12) as key components of peer group supervision. Our study identified Confidence in Practice (Questions 1,4,6,9), Team Support (Questions 2,3,5,10,14) and Group Safety (Questions 7,8,11,12,13). The internal consistency reliability measured by Cohen’s alpha for our factors were as follows: 0.78 (confidence in practice); 0.83 (team support); 0.59 (group safety). Confidence in Practice and Team Support showed good to high reliability, whereas Group Safety showed the lowest reliability. Overall, the responses were too variable to be statistically significant. The emphasis in our study’s findings was less on the PGS process and more on support rather than supervision (Table 2).

**Table 2:** Rotated factor matrix.^a^

	Factor		
	1	2	3
Team support			
Q2 I feel safe sharing workplace issues in peer group supervision sessions	**.871**	.094	.041
Q10 I feel confident about bringing issues to peer group supervision	**.740**	.116	.022
Q3 I believe that any confidences I share are respected	**.691**	.150	.022
Q5 There is mutual trust between the members in my group	**.634**	.160	.162
Q14 I am clear about what I want to get out of peer group supervision	**.341**	.269	.105
Group safety			
Q12 Peer group supervision has made me more aware of areas of skill I need to improve	‐.069	**.725**	.199
Q11 Peer group supervision has helped me cope with any stresses at work I may have	.335	**.627**	−.141
Q7 Being part of a peer supervision group is helping to develop my self‐awareness	.297	**.410**	.341
Q13 There are well established ground rules in my group	.349	**.401**	−.074
Q8 Peer group supervision has helped me feel more confident about dealing with my job	.209	**.333**	.175
Confidence in practice			
Q4 I have gained new clinical insights through peer group supervision	.066	.156	**.678**
Q6 Peer group supervision has definitely had a positive impact on the quality of care I provide	−.001	.299	**.669**
Q9 The purpose of peer group supervision is to enable practitioners to feel confident in their own practice	.168	−.049	**.406**
Q1 The purpose of peer group supervision is to improve patient care	−.028	−.014	**.253**

Extraction Method: Maximum likelihood.

Rotation Method: Varimax with Kaiser normalisation.

*Values in bold indicate the highest factor loading to each factor/component*

^a^ Rotation converged in 5 iterations.

### Findings from qualitative data analysis

Thematic analysis of data from the open‐ended question of each survey revealed four key themes and provided insight into the quantitative data. Supporting participant quotes for these themes are provided in Table 3.

**Table 3:** Supporting participant quotes for the four key qualitative themes.

Theme	Supporting quote
Theme 1: Being part of a supportive groups who ‘gets it’	*Our group was more established to help us work though our workplace difficulties and to help improve the emotional well‐being of staff members and allow them a safe place to communicate frustrations, concerns and struggles both in the workplace and personal. This was personally incredibly helpful personally as I have like‐minded people who acted as a sounding board who I knew had mutual respect and understanding.* *(Māori/6–10 yrs. service/21–29 y/o)*
	*Some of the more useful sessions stemmed from just checking in with each other…it was more about peer support than peer supervision. (16–20 yrs. service/40–49 y/o)*
Theme 2: Time‐out to reflect on practice and problem solve	*It is nice to share my experiences and problems and gain new insight into situations that I have trouble coping with or finding the best approach to manage a situation appropriately. (0–5 yrs. service, 21–29 y/o)*
	*This group has made me talk about myself more and my actions which would normally be ignored. I feel it has made me reflect on my practice more, and my interactions with staff. (6–10 yrs. service/21–29 y/o)*
	*I feel that I can take a bit of time to reflect on my practice before I go to peer support… having the peer support has made me aware there are other ways and to maybe seek a second opinion. (16–20 yrs. service/30–39 y/o)*
Theme 3: Organisational barriers	*Full attendance of my group is rare. Most months no one turns up. Mainly because patients are booked on the machines or work is over due [sic]. I feel peer supervision is not a priority and it should be. (16–20 yrs. service/40–49 y/o)*
Theme 4: Group process issues *Membership*	*Sometimes you have an issue with a member of your peer supervision group and it is hard to talk about within the peer supervision setting. I think it would be easier with a person who is an RT but not in your workplace. (6–10 yrs. service/21–29 y/o)*
	*The only thing I would like to improve is the trust issues. I have heard things come out of peer supervision which have been said in confidence. (16–20 yrs service/30–39 y/o)*
Theme 4: Group process issues *Structure*	*I have found that my peer group does not use the ground rules distributed as part of the peer group supervision project but have assimilated our own rules into our sessions. I feel that Peer Group Supervision in my group should probably be called Peer Group Support but all the same it has improved how I manage our patient workload which in turn must improve our patients experience in our centre. Therefore it has improved how I deal with patients albeit on a more strategic level as opposed to one on one interactions. (31+ yrs. of Service/50–59 y/o)*
	*I think our group would benefit from stronger leadership/facilitation as our last time was a real vent session – I do not feel that it was beneficial at all but it is difficult to communicate this to the group because I do not want to ‘put down’ the person who raised the issue. (16–20 yrs. service/30–39 y/o)*

1. **Being part of a supportive group who ‘gets it’**

Most RTs valued the opportunity to be part of a group where they felt safe, respected and supported and were able to discuss stressful workplace issues. It appeared that this helped to reduce feelings of isolation, nurture group trust and provide an outlet to offload so that challenging topics could be discussed in a safe environment. This was shared across all age, gender and ethnic groups, irrespective of years of practice.

- **Time‐out to reflect on practice and problem solve**

Most RTs appreciated having time out with colleagues to improve their practice. They valued being able to discuss and problem‐solve clinical and professional issues. However, several RTs also commented that PGS did not improve the quality of their care to patients.

- **Organisational barriers to peer group supervision**

A recurring issue across all groups was the challenge of prioritising time to meet regularly. Clinical workload pressures and variable rosters for part‐time staff meant that some groups met infrequently or not at all. This was evident across all centres.

- **Organisational barriers to peer group supervision**

*Membership*

While many participants enjoyed being in a safe and trustworthy group, this was not shared universally. Some participants indicated that trust within the group membership did not develop because of members' behaviour and dynamics: judgment, breaking group confidentiality, interpersonal conflict, lack of leadership‐sharing, differing expectations, venting, gossiping and fluctuating attendance. These behaviours did not foster a safe and supportive environment in which to discuss workplace issues in a constructive manner.

*Structure*

Several participants reported that groups were not facilitated well because of there was lack of clarity regarding group structure, including poor leadership. The purpose of the meeting, expectations of member behaviour, and co‐leadership were unclear to most participants except for RTs in their first five years of practice. As a result, the RT groups with more than five years’ experience often defaulted to venting or gossiping, and they were reluctant to take turns in leadership to keep the group on track.

## Discussion

This study aimed to investigate New Zealand RTs’ perceptions of participating in PGS. Overall, both quantitative and qualitative data indicate that RTs appreciated the time to meet with colleagues and generally felt comfortable to bring up clinical issues with their peer group. PGS gave the RTs the chance to discuss interpersonal aspects of radiation oncology care that could potentially lead to burnout. RTs in their first five years of practice appeared to discuss patient‐related stressors and they perceived these discussions to help their RT practice. The more experienced staff focussed on organisational‐related stressors. However, there was widespread consensus amongst participants that groups lacked structure, which diminished the effectiveness of PGS. Furthermore, lack of clarity about the purpose of PGS and expectations of members’ behaviour undermined some participants’ trust in the group, which contributed further to ineffective PGS meetings. This confusion meant that meetings became avenues for venting and gossiping which is not the purpose of PGS and resulted in several of the participants stating that PGS did not improve the quality of care they gave patients. This suggests that although reflection on practice was appreciated, participants did not readily transfer this reflection to patient care. It is evident from the data that RTs used PGS as a form of peer support rather than supervision. This is in contrast to the findings in the Horton et al.’s study.^18^

Previous research involving allied health professionals participating in PGS found similar results to Horton et al.'s purpose, process and impact of supervision, where staff reported improved practice and patient care.^11^, ^16^, ^17^, ^18^ These studies included allied health professionals, such as social workers, occupational therapists, nurses, physiotherapists, dietitians, speech therapists and podiatrists. These professional groups work one‐on‐one with patients and clients, rather than as a team, unlike RTs, who work predominantly in teams of two or more, when treating cancer patients. Teamwork is a safety measure to ensure that patients are treated accurately with staff checking each other’s planning, treatment set‐up, and other relevant checks before delivering radiation therapy to the patient. Therefore, RTs work closely with their colleagues and become familiar with how each member of the teamworks as staff have varying levels of experience and expertise. There is also a team leader who is responsible for the efficiency of each RT team. These small hierarchical RT teams may have had an impact on how the groups functioned. Firstly, working in small teams RTs get to know each other well so the findings from this study may be reflective of existing relationships within each RT centre. This may account for the diversity in responses to trust and safety issues, influencing group dynamics within some groups. Secondly, taking responsibility for leadership of a session seemed to be particularly challenging for RTs. This may be due to working in small teams as RTs may be reluctant to take on a leadership role because they work collectively rather than individually. Peer support may come more naturally than PGS since their work is often checked by a colleague.

Trialling multi‐disciplinary groups could be an alternative to the homogenous RT group. RTs would be mixed with other allied health professionals and therefore would not be familiar with everyone in their peer group eliminating the issues of working in small teams. Kuipers^17^ investigated if structured arrangements for allied health group supervision lead to better outcomes for peer group members. This study reported that multidisciplinary peer groups were rated as having similar impacts, processes and purposes as the more homogenous single‐discipline groups. The single‐discipline groups were individuals that did not necessarily work in the same institution. Future research will investigate whether RTs would respond better to structures and processes, such as assigning leaders and sticking to the group rules, in a multi‐disciplinary group with whom they are less familiar.

In the current study, staff within their first five years of clinical practice were more likely to be clear about the purpose of PGS, structure their meetings, discuss patient‐related issues and perceived improvements in their patient care. RTs with more than five years’ clinical experience particularly enjoyed the group process and support gained from their peers, rather than deepening or extending their work practice. They also were more likely to choose an informal approach to their PGS meetings, appreciating the time out to reflect and discuss work‐related issues in a safe environment. Peer support appeared to be especially important to this group, contributing to more positive workplace relationships and self‐awareness rather than developing more confidence in the quality of their patient care. Limited numbers of male and ethnic minority RTs make it difficult to interpret the findings for these sub‐groups. Given that there are low levels of male and ethnic minority RTs in the NZ workforce, these groups may be more appreciative of inclusive supportive collegial relationships.^3^, ^18^

These findings are consistent with those of the 2013 NZ study,^3^ in which radiation oncologists, radiation therapists, oncology nurses and physicists identified that high levels of ‘Occupational Stressors’ (patient‐related and organisational) were contributing towards burnout. For senior RTs, it was organisational stressors, such as, dysfunctional staff dynamics, perceived poor management, low staff morale and a lack of career progression that were their trigger points for burnout, and not patient‐related stressors. This finding is not surprising as workplace culture becomes increasingly important as practitioners progress along their career pathway.^19^ Conversely, for RTs in their first five years of experience, it was the patient‐related stressors, such as, young patients, people their own age and palliative care patients, that were potentially causing them stress. This is to be expected as RTs with less working experience are often less confident in managing patient care at the start of their career and grow in confidence along their career pathway.^3^, ^7^, ^20^ For this reason, PGS has been introduced into the Bachelor of Radiation Therapy (BRT) programme at the University of Otago, Wellington campus, so that students will be familiar with the process when entering the workforce. Research into the effectiveness of PGS for students will be carried out over the next five years.

## Limitations

Although there were representatives from each centre at the two training workshops, not everyone who participated in PGS was able to attend. The responsibility then fell on the attendees to report back to their respective group members/centres on their learning. This could have had an impact on how practitioners set up PGS in terms of structure and purpose if information was not communicated correctly. However, misunderstandings about structure and process did not appear to change after the in‐service training between the surveys. In future, it would be recommended that all staff have formal training.

All of the data have been collected via RT self‐report. There could be environmental factors such as completing the survey at work or recent negative or positive experiences of PGS that may have influenced their recall.

Finally, while the quantitative results suggested some overlap in identified factors with those reported by Horton et al.^18^, some items did not correspond to the same groupings between studies. This lack of confirmation may be a consequence of sample characteristics (in terms statistical variability, partially related to the size of the two studies and hence statistical precision) or the nature of the study groups (given the substantively different target groups in the two studies: radiation therapists vs. speech/language therapists) and hence broader context around relevant dimensions affecting coping.

## Conclusion

Overall, RTs responded positively to PGS. This study indicates that PGS maybe useful in reducing burnout for RTs in their first five years of practice. They perceive PGS as a way to improve their patient care and reduce their stress because they feel they can discuss patient‐related matters at the meetings. In contrast, more experienced staff are using the groups as a ‘professional support group’, rather than ‘PGS’, as a strategy for managing the organisational stressors associated with burnout. Further research will examine the effectiveness of introducing PGS to students and trialling multi‐disciplinary groups in the oncology setting.

## Conflict of Interest

The authors declare no conflict of interest.
